# GNSS Positioning by CORS and EGM2008 in Jilin Province, China

**DOI:** 10.3390/s151229806

**Published:** 2015-12-04

**Authors:** Qiong Wu, Jingyu Kang, Shuwen Li, Jianing Zhen, Hongqing Li

**Affiliations:** 1 College of Geo-exploration Science and Technology, Jilin University, Changchun 130026, China; wuqiong@jlu.edu.cn (Q.W.); kangjy14@mails.jlu.edu.cn (J.K.); zhenjn13@mails.jlu.edu.cn (J.Z.); 2 Jinan Institute of Survey and Investigation, Jinan 250013, China; lishuwen90@gmail.com

**Keywords:** continuously operating reference station, EGM2008, geoid, ellipsoid height, CGCS2000, coordinate transformation

## Abstract

The Continuously Operating Reference Station (CORS) technique has been widely applied in land resource management, surveying, mapping, deformation monitoring, precise navigation, *etc.* This article analyzed the positioning method using EGM2008 and CORS of Jilin Province, China. The vertical transformation of EGM2008 from WGS84 to China’s CGCS2000 datum and the horizontal coordinate transformation from CGCS2000 to a triangulation coordinate system were discussed. The results indicated that a local geoid with respect to CGCS2000 can be transferred from EGM2008 with the same accuracy, and the geoid correction between CGCS2000 and WGS84 varied from 0.023 m to 0.111 m. The coordinate transformation method based on the curve surface approximation method indicated that the theoretical error was less than 0.09 m in the grid within 10° longitudinal and 5° latitudinal, and less than 0.3 m in large area and 0.1 m in small area in field validation. The method proposed in this article expanded the positioning result and its application for JLCORS and other CORS with local datum.

## 1. Introduction

GNSS has been widely applied in point positing, navigation, deformation, Earth gravity studies, water vapor tomographics, *etc.* [1,2]. A Continuously Operating Reference Station (CORS) system lays out a GNSS reference with a certain density by tracking satellite signals continuously, which can provide carrier phases and code measurements transferred to rover stations by network communication technology to support three dimensional positioning with centimeter accuracy [3,4]. 

CORS has been established in many countries and areas. CORS maintained by the U.S. National Geodetic Survey (NGS) provide services such as carrier phase, pseudo-range observation and other GNSS data to support three-dimensional positioning, meteorology, and geophysical related applications throughout the United States and some other countries with centimeter-level to millimeter-level precision in the National Spatial Reference System [5]. The Canadian Spatial Reference System (CSRS) was established and managed by Natural Resources Canada (NRC), and the core of CSRS includes the Canadian Active Control System (CACS) and a continuously operating GNSS receiver network [6]. CSRS provides users with millimeter-level positioning services in support of mapping, marine charting, boundary demarcation, crustal deformation study and other georeferencing applications [6]. In Germany, Satellitenpositionerungsdienst (SAPOS) unified differential GPS in various departments, which include more than 200 permanent GPS reference stations, and provides centimeter-level real-time positioning services as well as millimeter-level high-accuracy positioning services. Other European countries have also established permanent GPS reference stations with similar functions as the national geographic information benchmark, and provide services including GPS differential positioning, navigation, deformation, *etc.* [7]. In Asia, The Geographical Survey Institute of Japan (GSI) established in 1993 the GPS observational network, which possesses more than 1200 continuous GPS stations with a mean separation distance of 25 km covering the whole country, and can be applied in crustal deformation monitoring, prediction of earthquakes, meteorology, GPS real-time positioning, *etc.* [8]. 

China’s first CORS was the Shenzhen Continuous Operational Reference System (SZCORS) established in 2003 by the local government of Shenzhen, in Guangdong Province. SZCORS includes five GNSS continuously operating reference stations and a local geoid of 1 cm accuracy, and provides positioning services (3 cm on horizontal and 5 cm on vertical) in support of geodesy, engineering surveying, meteorological monitoring, earthquake monitoring, precision navigation, *etc.* [9]. Since then Beijing, Tianjin, Shanghai, Chengdu, Chongqing, Kunming, Wuhan, Hong Kong and some other cities also established CORS [10].

The Earth Gravitational Model 2008 (EGM2008) was released by the EGM Development Team of the National Geospatial-Intelligence Agency (NGA). It is a high order gravitational model complete to spherical harmonic degree and order 2159, while coefficients extending to degree 2190 and order 2159 was also included [11,12,13]. The overall accuracy of EGM2008 in China is about 20–24 cm in Western China, 12 cm in Mid-east China, and 9 cm in North China [14,15].

**Figure 1:**
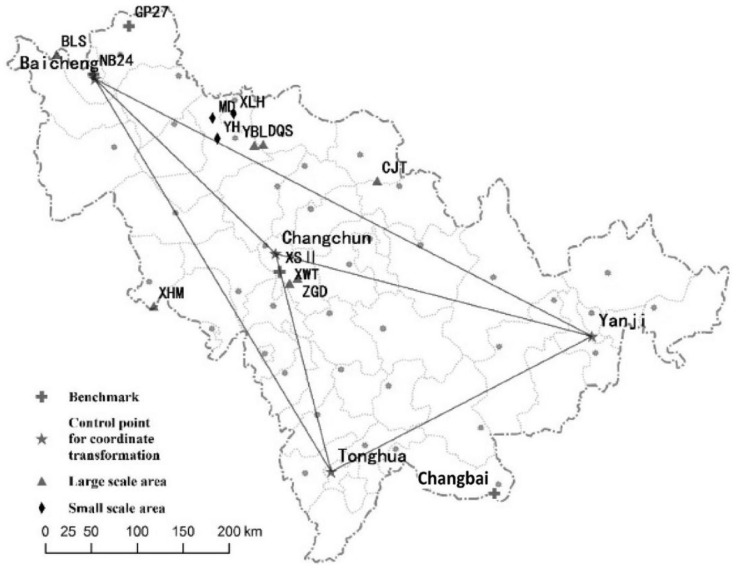
A map of the study area.

Jilin Province is located in Northeast China. The terrain inclines from southeast to northwest, with mountains in the eastern part and plains in the middle and western part. The average elevation is 260 m (Figure 1). The Jilin Continuously Operating Reference Station (JLCORS) was established by the Bureau of Surveying Mapping and Geoinformation of Jilin Province. JLCORS consists of 49 reference stations covering the entire Jilin Province (121°37′-131°18′ E, 40°51′-46°19′ N, 187,400 square kilometers), and the present service aims to provide high accuracy (2–3 cm horizontal and 4–5 cm vertical) coordinates with CGCS2000 datum [16,17,18]. Virtual Reference Station (VRS) technology was applied in JLCORS, and consists of four major sections including a continuous GPS reference station network, data processing center, data communication section and user section [19], which integrates internet technology, wireless communication technology, computer networks and GPS positioning technology. JLCORS provides coordinates within CGCS2000, while many users need the coordinates with respect to old coordinate systems based on triangulation technology such as Beijing54 or Xi’an80 datum [20,21,22], which means the conversion problem from CGCS2000 to these two old datum formats needs to be solved. Another issue that needs to be solved for the application of JLCORS is the definition of local geoid, which is independent in the height conversion from ellipsoid to normal height or orthometric height (the difference of these two height systems is too small to discriminate considering the 200 m average height of the study area). This article studied the coordinate transformation model from geocentric CGCS2000 to the old reference ellipsoid systems (Beijing54 or Xi’an80), and calculated the local geoid based on EGM2008. The accuracy of coordinate transformation and the local geoid was analyzed using field data, and the application of the proposed method is discussed.

## 2. Methods 

### 2.1. Local Geoid Calculation Based on EGM2008 

The Earth gravity model provides the information to transfer the ellipsoid height to orthometric height or normal height [23,24]. The spherical harmonic function expansions of geoid undulation can be expressed as [25,26,27,28,29]:

(1) $$N=R\overset{n_{max}}{\underset{n=2}{\sum }}\overset{n}{\underset{m=0}{\sum }}\left({\bar{C}}_{nm}Cos\;m\lambda +{\bar{S}}_{nm}Sin\;m\lambda \right){\bar{P}}_{nm}\left(Sin\;\varphi \right)$$

where $n_{max}$ is the highest order of the expansion, ${\bar{C}}_{nm}$ and ${\bar{S}}_{nm}$ are the full normalization coefficients, λ is the geodetic longitude, φ is geodetic latitude, and *R* is the Earth’s average radius.

**Figure 2:**
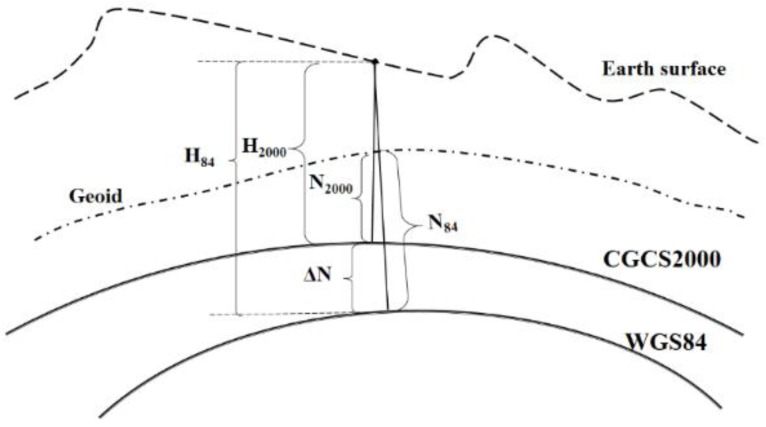
Geoid bias between CGCS2000 and WGS84.

Two kinds of bias need to be corrected to convert the EGM2008 geoid from WGS84 to CGCS2000. One is the constant gravity datum difference between China and EGM2008, the other is the ellipsoid difference between WGS84 and CGCS2000 (Figure 2).

The constant bias of vertical datum between China and EGM2008 can be expressed as:

(2) $$∆h=H_{84}-h-N_{84}$$

where $H_{84}$ is the ellipsoid height of WGS84, h is the orthometric height, $N_{84}$ is the geoid undulation of EGM2008 with respect to WGS84.

The ellipsoid datum difference between WGS84 and CGCS2000 can be expressed as:

(3) $$∆N=H_{84}-H_{2000}$$

where $H_{2000}$ is the ellipsoid height with CGCS2000.

The corrected geoid undulation within CGCS2000 is expressed as follows:

(4) $$N_{2000}=N_{84}-∆N+∆h$$

The correction method for ∆h is calculated through the observation of precise levelling points measured by static GPS observation within ITRF2008, and this constant is 0.293 min for Jilin Province [27]. To get the correction of the systematic bias from different coordinate systems, a Trimble GNSS R8 receiver was used by synchronous static observation (8 h) in Changchun, Tonghua, Baicheng and Yanji, which nearly covered the entire province (Figure 1). The coordinates with CGCS2000 of these points was also measured per hour. The static observation data obtained from these four GPS receivers was calculated in ITRF2008. Transformation parameters from ITRF2008 to CGCS2000 were calculated by the Bursa model. Coordinates of grid points at the surface of WGS84 (zero ellipsoid) was transferred to coordinates in CGCS2000 by a 7-parameter datum transformation model to get the ellipsoid difference between the two datum in CGCS2000:

(5) $$∆N=0-H_{2000}$$

where$\;H_{2000}$ is the ellipsoid height within CGCS2000 transferred from ellipsoid surface with respect to WGS84. The errors of geoid due to the value difference of coordinate with respect to the two geodetic datum can be neglected because this difference is very slight (Table 1). 

**Table 1:** The horizontal difference (UTM Projection) of geodetic coordinates between WGS84 (ITRF2008) and CGCS2000 (m).

Point Name	dx	dy
Tonghua	0.154	−0.435
Baicheng	0.178	−0.431
Yanji	0.166	−0.437
Changchun	0.153	−0.462

### 2.2. Coordinate Transformation

The 7-parameter datum transformation model was applied to transfer coordinate from CGCS2000 to Beijing54 or Xi’an80:

(6) $$\left[\begin{array}{c} X_{\alpha }\\ Y_{\alpha }\\ Z_{\alpha } \end{array}\right]=\left[\begin{array}{c} ∆X\\ ∆Y\\ ∆Z \end{array}\right]+\left(1+k\right)\left[\begin{array}{c} X_{\beta }\\ Y_{\beta }\\ Z_{\beta } \end{array}\right]+\left[\begin{array}{ccc} 0 & \varepsilon_{Z} & -\varepsilon_{Y}\\ -\varepsilon_{Z} & 0 & \varepsilon_{X}\\ \varepsilon_{Y} & -\varepsilon_{X} & 0 \end{array}\right]\left[\begin{array}{c} X_{\beta }\\ Y_{\beta }\\ Z_{\beta } \end{array}\right]$$

where $X_{\alpha }$, $Y_{\alpha }$, $Z_{\alpha }$, $X_{\beta }$, $Y_{\beta }$ and $Z_{\beta }$ represent the geodetic rectangular coordinates of corresponding points in different coordinate systems, while ∆X, ∆Y, ∆Z, $\varepsilon_{X}$, $\varepsilon_{Y}$, $\varepsilon_{Z}$ and *k* are the seven parameters used in the transformation between different coordinate systems [30,31,32].

This model needs to input the geodetic coordinates of both coordinate systems, while the Beijing54 or Xi’an80 datum was established by triangulation technology, so it is impossible to get a high precision ellipsoid height, which means the rectangular space coordinate has a low accuracy, and it is not feasible for a relatively high accuracy transformation [33,34]. The target for the application of this model is to transfer the horizontal coordinates from CGCS2000 to the local ellipsoid, so in order to apply the model for horizontal coordinate transformation, we modified the input data and the algorithm of the Bursa model based on the curve surface approximation. The CGCS2000 ellipsoid and local ellipsoid are geometrical similar spatial curved surfaces for a relatively small area, and the transformation between the two datum in this small area can be defined as the spatial curve surface approximation process from one ellipsoid surface to another ellipsoid surface. Because the form of both ellipsoids was defined, three common points with the same horizontal coordinates in both of the datum are the minimum point amount needed for the approximation. The algorithm is to set the ellipsoid height as zero for both the CGCS2000 and triangulation local datum firstly, and the equation was solved by an iteration method. The process replaces the geodetic height both in CGCS2000 (H_α_) and Beijing54 (H_β_^0^) with 0 before the parameter solution, next, it translates Hα to the geodetic height in Beijing54 (denoted by H_β_^1^) with the use of the seven parameters above, let ${\text{d}}_{1}=\sum {\left({\text{H}}_{\text{α}}-{\text{H}}_{\text{β}}^{1}\right)}^{2}$, and then, use ${\text{H}}_{\text{β}}^{1}$ to solve the seven parameters again to get the new geodetic height in Beijing54 (denoted by ${\text{H}}_{\text{β}}^{2}$), let ${\text{d}}_{2}=\sum {\left({\text{H}}_{\text{α}}-{\text{H}}_{\text{β}}^{2}\right)}^{2}$, and the iteration should continue until ${\text{d}}_{2}\geq {\text{d}}_{1}$. 

This method does not have the general spatial seven parameters significant for datum transformation, they are the parameters for the approximation of the ellipsoid surface in the study area. In fact, the ellipsoid height of common points are different in the two coordinate systems, and this difference value was calculated and used to measure the approximation level of the two ellipsoid surfaces during the iteration process. This algorithm does not change the shape or the curvature of the ellipsoid surface, which means that the common points in two ellipsoids cannot totally overlap and there are errors in the common points, and these errors can be attributed as the model error. The model errors change with different ellipsoid parameters or study areas, and can be calculated based on the ellipsoid parameters (Table 2). The curved surface approximation make it possible to calculate transformation parameters in a situation lacking Beijing54 geodetic height data in the application of the Bursa model. In a large area (longitudinal span is 9°, and latitudinal span is 4° in middle latitude zones), the theoretical model error is less than 9 cm.

**Table 2:** The calculation errors of both theoretical and field data (CGCS2000 to Beijing54).

Data Source	Errors	Large Zone	Middle Zone	Small Zone
		122°–131° E	122°–123° E	124°30′–124°45′ E
		41°–45° N	43°–45° N	45°00′–45°15′ N
Theoretical	Ellipsoid height	0.016 m	0.004 m	0.000 m
	Horizontal	0.090 m	0.020 m	0.005 m
Field	Ellipsoid height	No data	0.016 m	0.004 m
	Horizontal	No data	0.300 m	0.100 m

Errors in the common points lead to the oscillation of ellipsoid height correction values in small range. The iteration stopped when d_1_ < d_2_, that is, the stop condition of the iteration is the beginning of the oscillation. The approaching of the two ellipsoid surface in the calculation process can be attributed as the distance minimization of common points on the two ellipsoid surfaces. When the iteration stopped, the two ellipsoid surfaces can be attributed as approached by the distance minimization of common points and these distances approach the average vertical distance between the two ellipsoid surfaces at these common points.

## 3. Results

### 3.1. Geoid Calculation with CGCS2000 

#### 3.1.1. The Calculation of Transformation Parameters

We applied 7-parameter datum transformation model to calculate the seven parameters which are used to calculate ∆N. Three field measurement points (Tonghua, Baicheng and Yanji, Figure 1) were set as control points, Changchun (Figure 1) was set as checkpoint during the transformation between CGCS2000 coordinates and WGS84 coordinates. The conversion accuracy of each point is shown in Table 3.

**Table 3:** Accuracy of transformation parameters (m).

	Point Name	Errors	
		Horizontal	Vertical
Control Point	Tonghua	0.006	0.027
	Baicheng	0.004	0.030
	Yanji	0.004	0.028
Check point	Changchun	0.005	0.029

The data showed a good compatibility between CGCS2000 and ITRF2008. Statistics of the eight measured values (measurement interval is one hour) in every point by JLCORS shows that the maximum error in x, y was 0.016 m, 0.007 m, respectively, and corresponding RMS was 0.006 m, 0.003 m, which proved the stability of JLCORS.

**Figure 3:**
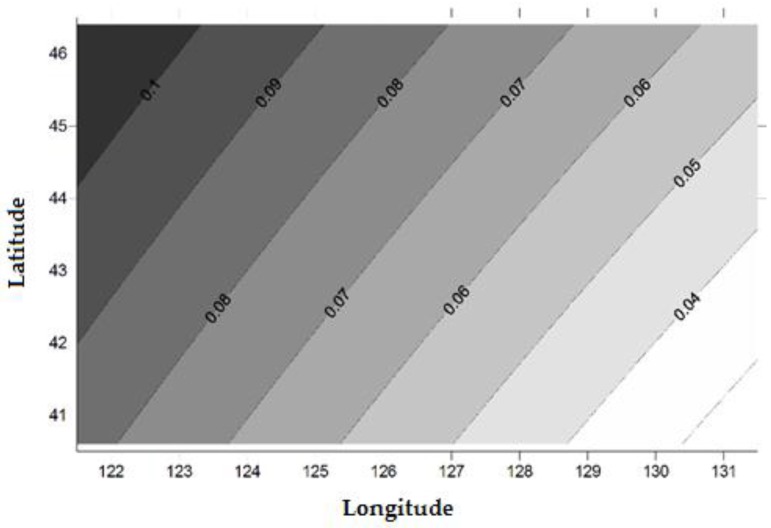
Ellipsoid datum difference between WGS84 and CGCS2000 in Jilin Province (m).

**Figure 4:**
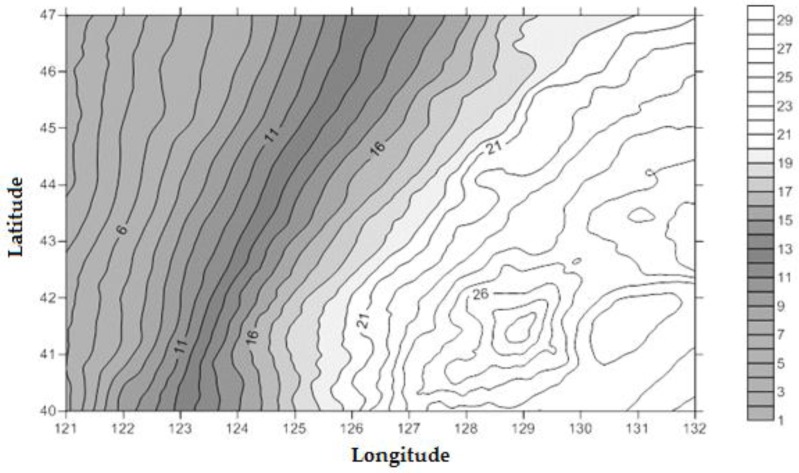
Geoid undulation in Jilin Province with respect to CGCS2000 (m).

The systematic biases (∆N) between WGS84 and CGCS2000 obtained by the coordinate transformation increased from southeast to northwest in Jilin Province. The minimum value is 0.023 m, the maximum value is 0.111 m and the average is 0.068 m (Figure 3), and the Geoid undulation of Jilin Province with respect to CGCS2000 is shown in Figure 4.

#### 3.1.2. The Validation of Geoid of Jilin Province with Respect to CGCS2000

Four uniformly distributed GPS leveling points in the plain area and one GPS leveling point in a mountain area (Figure 1) of Jilin Province was used to validate the calculated local geoid, and the result is listed in Table 4. The general accuracy is less than 0.030 m in the plain area and 0.050 m in the mountain area.

**Table 4:** The validation of the local geoid with respect to CGCS2000 (m).

	Point Name	Orthometric Height		∆*h*
		Benchmark	Measured by CORS and Local Geoid	
Plain area	YH	139.700	139.721	−0.021
	NB24	155.078	155.105	−0.027
	GP27	135.623	135.601	0.022
	XSⅡ	231.008	231.011	−0.003
Mountain area	Changbai	729.315	729.363	0.048

### 3.2. Coordinate Transformation from CGCS2000 to Local Geodetic Coordinate

Using the curved surface approximation method, two scales of areas in Jilin Province were selected to calculate the transformation parameters from CGCS2000 to Beijing54, and the resulting transformation accuracy is shown in Table 5. The results indicate that for a large area (50,000 square kilometers), the horizontal errors are less than 0.300 m, while for small areas (250 square kilometers), the horizontal errors are less than 0.100 m, which indicates that the proposed method has fine suitability for the coordinate transformation.

**Table 5:** The accuracy of coordinate transformation from CGCS2000 to Beijing54 (m).

Area	Point Name	Horizontal Residuals
Large scale area	YBL	0.288
	BLS	0.293
	CJT	0.129
	ZGD	0.248
	XWT	0.138
	XHM	0.225
	DQS	0.280
Small scale area	XLH	0.045
	MD	0.098
	YH	0.072

## 4. Discussion and Conclusions

The Earth Gravitational Model 2008 is a geoid model that has a good adaptability in the plain areas of Northeast China, with high accuracy on a global range. The maximum error in CGCS2000 was 0.030 m and the mean square error was 0.022 m after the system deviation and constant deviation were added to the model. The results showed that it is an efficient and powerful method for improving the accuracy and reliability of GPS height measurements by JLCORS.

The results of applying the transformation algorithm between the CGCS2000 and Beijing54 coordinates showed that the use of the surface approximation method to calculate the seven two-dimensional parameters is a highly effective and simple away of obtaining objective coordinates under conditions of unknown or low accuracy geodetic elevation. Transformation parameters should be calculated by partition if accuracy is highly expected for a large area.

WGS84 coordinates have good compatibility with the CGCS2000 coordinate system adopted in JLCORS that runs 24 h a day without break. With better observation conditions and in the signal coverage, rover stations can achieve high location accuracy utilizing one GPS receiver. In addition, coordinates of object points in different coordinate systems can be rapidly obtained by coordinate transformation, and orthometric height or normal height can also obtained quickly with the application of local geoids. With their characteristics of high efficiency, high accuracy, high reliability and low cost, CORS will play a more significant role in the field of control survey, cadastral survey, land management, water resource management, urban planning, line planning, field data collection, topographic mapping, point laying-out, *etc.*
